# Worth the Wait? The Effect of Comparative Framing on Tourists’ Waiting Intention

**DOI:** 10.3390/bs16020167

**Published:** 2026-01-25

**Authors:** Jun (Justin) Li, Shuaifang Liu, Yiyan Wang, Nuo Dong, Yingshan Guo, Woo Gon Kim, Qinglei Cai

**Affiliations:** 1 School of Tourism Management, South China Normal University, Higher Education Mega Center, Guangzhou 510006, China; justinli83@scnu.edu.cn (J.L.); 2023023722@m.scnu.edu.cn (S.L.); 2024023954@m.scnu.edu.cn (Y.W.); 2025023488@m.scnu.edu.cn (N.D.); 20221132030@m.scnu.edu.cn (Y.G.); 2 Faculty of International Tourism and Management, City University of Macau, Taipa, Macau; 3 International Center for Hospitality Research & Development, Dedman College of Hospitality, Florida State University, 288 Champions Way, UCB 4115, Tallahassee, FL 32306, USA; wkim@dedman.fsu.edu; 4 School of Health Management, Southern Medical University, Baiyun District, Guangzhou 510515, China

**Keywords:** comparative framing, waiting intention, perceived waiting costs, prospect theory, queuing

## Abstract

Queuing is almost inevitable in tourist service experiences, but most tourists are reluctant to wait. Drawing on prospect theory, this study examined how comparative framing influences tourists’ waiting intention. Across three scenario-based experiments, the research found that, compared with non-comparative framing, comparative framing can effectively enhance tourists’ waiting intention. Perceived waiting costs play a mediating role in the impact of the comparative framing on waiting intention. Additionally, the queuing settings play a moderating role, and the mediating effect is stronger in physical queues than in virtual queues. This research shifts the analytical focus from objective waiting time to the framing of waiting-time information, reveals a psychological cost assessment mechanism based on reference points, and enriches the theoretical explanation of tourists’ immediate decision-making in tourism services. It also provides practical references for optimizing service information and queue management during peak hours.

## 1. Introduction

In recent years, the global tourism market has continued to expand, and the number of tourists has repeatedly reached a record high. According to the latest UNWTO World Tourism Barometer, international tourist arrivals reached 1.4 billion in 2024 (UNWTO, 2025). Queuing in theme parks, popular scenic spots, and large-scale festivals is becoming more and more common. Although a long queuing time does not necessarily have negative consequences (Ryan et al., 2018; J. Kim et al., 2025), too long a queuing time still consumes the precious time of tourists, and is often accompanied by negative emotions such as anxiety, fatigue, and uneasiness. The overall experience of tourists even affects the safety, order, and operational efficiency of scenic spots (S. Li et al., 2021). The negative emotions generated in tourists during the waiting process not only reduce their satisfaction with the scenic spot, but also may affect their future choices and loyalty (H. Lin et al., 2020; Im & Seo, 2024). However, during peak periods, traditional approaches, such as capacity expansion, additional staffing, and spatial modifications, show diminishing returns, given their high costs and limited scalability. Moreover, due to the pronounced seasonality of tourism (D. Zhang & Xie, 2023), aggressive capacity expansion solely to shorten wait times during peak periods often leads to inefficient resource allocation and poor cost-effectiveness. This raises an urgent question: can tourists’ acceptance of queues be improved through psychological and behavioral interventions?

Despite the broad research focus on tourists and queuing experience, the current studies mainly focus on emotional regulation and the alleviation of adverse consequences (such as anxiety, boredom, and decreased satisfaction) during the queuing process (Efrat-Treister et al., 2019). Much less attention has been paid to the pre-queuing phase, which involves how tourists anticipate waiting times and make decisions. In fact, tourists have a tendency of calculating the waiting time (Lyu & Hwang, 2021), experience value, and the psychological cost when determining whether to join a queue. This is a mental process at the pre-queuing decision-making level that is of significance in comprehending their response regarding the behavioral aspect and general experience they have. The previous literature has mostly focused on variables such as the length of the queue (Weiss & Tucker, 2018) and the physical setting (Bonnin & Goudey, 2025), and there is little discussion on framing effects in the display of wait times. Individuals’ information-processing not rational at all times, and the phenomenon of framing has been long-standing in decision research (Alnes & Haugom, 2024; Wallach et al., 2025). This study adopts prospect theory as the core theoretical framework, emphasizing that individuals make decisions based on relative evaluations with respect to psychological reference points, rather than solely relying on absolute values (Wilms et al., 2020). Building on this, comparative framing provides explicit reference information, such as whether the current waiting time is below average, which could allow tourists to perceive waiting as a relatively favorable choice, thereby alleviating anxiety and enhancing their willingness to queue. Although a comparative framing role in consumer decision-making was first investigated in some marketing and service studies (Guha et al., 2018), very little information is available on its role in tourism queuing situations. Thus, the initial research objective is to investigate the impact of comparative framing strategies on the waiting intention of tourists.

Whether tourists are willing to wait for a service will always be based on their determination of whether the wait is worth it, which is not wholly based on objective time but also on the perceived cost on the psychological factor (Su et al., 2022). This mediating effect can be attributed to the social comparison theory, which states that people are more prone to changing their emotions and behavior expectations in reference to other parties through a process of comparison (Zhou & Soman, 2003), which explains how comparative framing affects the assessment of perceived costs of waiting. The existing literature has identified a relationship between the perception of the waiting cost and the reactions of consumers to the waiting experience (Jo et al., 2025). Compared with the absolute duration, factors such as the relative waiting position, the waiting time of others, and the scarcity of the current situation are often used as references to assess the cost customers have paid (Zhou & Soman, 2003), which influences the reaction of an individual and whether they will persist in staying in the queue.

Individuals’ perception of waiting costs does not remain consistent in all scenarios (Antonides et al., 2002). In the tourism industry, the diversity of queuing forms is increasingly prominent, especially with the rise in virtual queuing and physical queuing, which provide tourists with a completely different waiting experience. Virtual queuing allows tourists to move freely and carry out other activities while waiting, which often weakens attention to time clues. In contrast, physical queuing means that tourists are confined to a specific space (Djelassi et al., 2018), their physical discomfort and psychological anxiety are more obvious (L. Li et al., 2025), and their awareness of the waiting time is also stronger. Cognitive load theory suggests that individuals have limited cognitive resources. These different queuing forms activate different cognitive channels, which in turn affect an individual’s sensitivity to information cues. In a high-perceptual-load environment, individuals will prioritize information that is highly relevant to their current goal. Therefore, tourists in physical queues are more sensitive to the comparison framing of waiting time and are prone to a sense of relative advantage. However, in virtual queuing, this effect may be weakened. Therefore, this paper examines how queuing settings moderate the framing effect.

Building on these insights, this paper integrates prospect theory and cognitive load theory to systematically examine the influence mechanism and boundary conditions of the comparative framing of queueing time on tourists’ waiting intention. The rest of this paper is structured in the following way: Section 2 reviews the relevant literature on queuing, framing effects, and prospect theory. Using these theoretical frameworks, Section 3 formulates four hypotheses. Section 4, Section 5 and Section 6 report three scenario-based studies, including the samples, procedures, analyses, results, and brief study-level discussions. In Section 7 and Section 8, the key findings are summarized and the theoretical contributions, practical implications, research limitations, and future directions of study are discussed.

## 2. Literature Review

### 2.1. Queuing

Waiting can occur in any situation where service demand exceeds immediate supply. Whether in restaurants, scenic spots, or retail stores, long waiting times can easily lead to feelings of boredom, unfairness, and anxiety (Yin et al., 2024a). For example, when queuing for tickets or attractions at popular tourist sites, visitors often experience anxiety and dissatisfaction due to prolonged waiting. Such negative feelings can be ascribed to bad on-site management, resulting in a poor assessment of the traveling experience on the whole and the service brand (Chen et al., 2021).

The comparatively low degree of resources has led to studies concentrating on the role of strategic intervention in mitigating the dissatisfaction of queuing clients and their time pressure. Three main aspects are primarily the focus in this kind of research. Firstly, some studies aim to achieve a reduction in actual waiting time through process efficiency (Kambli et al., 2020), including aspects related to enhancing ticketing experiences in attractions, dining place-ordering services or hotel check-in services. Secondly, others try to keep the customers in a state of distraction and redirect their mind from the time that is passing by (Ding & Kyung, 2025). As an illustration, so-called display boards, installations, or performances are frequently presented in the queuing zone to distract tourists (Xu & Ding, 2023; Yu et al., 2024). The use of these environmental intervention measures suggest that that when customers’ attention is drawn by other stimuli, the subjective waiting time will be shortened. Thirdly, some studies focus on managing customer expectations by improving the presentation of service information. This includes clearly displaying estimated wait times, adding progress indicators, and notifying customers in advance about different waiting stages (Weiss & Tucker, 2018). This type of strategy is based on control theory, emphasizing that the “right to know” can enhance customers’ sense of control over the waiting process, thereby alleviating anxiety. Overall, these strategies use from multiple dimensions, such as environment, information and emotion, aiming to reduce the adverse consequences of waiting and enable customers to have a more positive subjective evaluation of the waiting process. However, no research has focused on the framing effect of queuing information at present.

Meanwhile, with the popularization of digital technology, virtual queuing systems have gradually emerged and become a new focus of waiting experience research in recent years. Compared with traditional physical queuing, virtual queuing allows customers to register online and freely arrange their waiting time without having to queue in person (Kyritsis & Deriaz, 2019). This model has been widely adopted in multiple service scenarios, such as catering, healthcare, and scenic spots, and is regarded as an effective means to enhance customer experience and optimize resource allocation. However, relatively few studies have focused on this new type of queuing method.

### 2.2. Framing Effect

The essence of the framing effect is that the contextualization of information presentation’s main mechanism is activating various psychological points of reference, thereby leading to systematic deviation (Fu et al., 2018). Classic examples include “90% survival rate” and “10% mortality rate”. Despite the objective probability being, similar people opt to make choices using the positive framework because the elements are expressed in a different way. Positive expression tends to recall positive feelings, whereas negative expression increases risk perception. This effect is not only a representation of the influence of loss aversion, but also an act of mental accounts and emotional arousal, and shows the strong impact of linguistic expression on cognition and judgment (Grolleau et al., 2016).

The research on framing effects mainly focuses on typical types of framing. One is goal framing, which explores the impact of information presentation regarding risk-based decision-making in the form of a loss or gain (Pang et al., 2025; Xue et al., 2024). The second is attribute framing, which emphasizes the positive and negative expression of individual attributes in the information, such as “hedonic vs. utilitarian”, which mainly affects consumers’ evaluation of products (Her & Seo, 2017; Yan et al., 2024). Although these studies extensively explore the influence mechanism of positive and negative expression, their focus is on different descriptions of a single object, lacking a systematic investigation of the comparison between options.

Compared with the above-mentioned traditional framing, this article focuses on comparative framing, which promotes individuals to make explicit comparisons between two or more options through information presentation. The core feature of comparative framing is relativity, and its effect comes from the relative position between options, not a single expression direction (Ye et al., 2021). Studies have found that social contrast tendency stimulates social anxiety, while time contrast helps self-improvement (Dagogo-Jack, 2024). In queuing contexts, when deciding whether to join a line, tourists need to psychologically evaluate the “costs and benefits” of waiting. By comparing the current waiting time with that of others or with a reference standard, tourists can establish a clear psychological benchmark, which alleviates anxiety and uncertainty and enhances their willingness to wait. However, the underlying mechanisms of comparative framing remain underexplored, particularly in tourism service contexts. Building on the unique mechanisms and practical relevance of comparative framing, this study examines its role in tourists’ queuing decisions, thereby extending the research on framing effects.

### 2.3. Prospect Theory

Prospect theory emphasizes the reference dependence of decision-making and presents asymmetrical value functions on the side of loss and gain. To be more precise, the value function is steeper during the loss interval than during the gain, which is an indication of loss-aversion. Simultaneously, the function demonstrates a fringe diminishing capacity, which is the diminishing returns impact (Tversky & Kahneman, 1992; G. Lin et al., 2024). In addition, prospect theory establishes a weighting mechanism of probability to understand why individuals overrate low-probability events and under-rate the medium–high-probability events (Higgins & Liberman, 2018). This theoretical model shows that there is a systematic deviation in human decision-making.

The current literature has used prospect theory to explain the impact of positive and negative framing on individual decision-making, and the impact of presenting information as a loss or a gain on risk inclination (Liu et al., 2023). However, reference dependencies are not confined to the negative and positive representation of an option. The psychological nature of referencing will also lead to relative comparisons that will influence the perception of value among individuals. Relative comparison helps people to influence their psychological anchors and comparatively determine values. When alternative choices are considered in a judgment, people will re-determine gains or losses depending on very clear comparison benchmarks, thus creating new reference standards (Head & Bruchmann, 2019). Comparative framing is different to traditional positive and negative framing. Nonetheless, it is also based on the concept of reference dependence in prospect theory, such that the subjective value of an outcome is based upon its relative location to a reference point.

From the perspective of prospect theory, external reference points offer definite psychological reference when consumers are faced with comparative framing. Such benchmarks enable people to make a more exact assessment of the costs and benefits of waiting (Schlotzhauer et al., 2025). By comparison, under non-comparative framing, individuals are forced to use their internal memory or personal experience, creating comparatively ambiguous reference points and diminishing the specificity of their evaluations.

## 3. Hypothesis Development

### 3.1. The Effect of Comparative Framing on Waiting Intention

Information presentation can have a marked influence on a person’s cognitive and behavioral reaction towards the same facts (Rahman et al., 2018; Wen et al., 2021). Prospect theory holds that people will make relative evaluations and operate around their psychological reference point to find the best option for the situation at that particular time (S.-J. Park & Yi, 2025). Thus, the presence of reference benchmarks in the information can contribute greatly to the subjective perception of a source and the behavioral inclination of people responding to the same information (Song et al., 2022).

In the case of queuing, at the pre-queuing decision stage, tourists have to make a mental trade-off between the cost of waiting and the benefits that might be incurred. Tourists do not have clear absolute criterion to use when presented with waiting time information; hence, they tend to apply empirical tests or situational indicators to determine whether to tolerate the waiting time or not. For example, when the waiting time is only presented in absolute values (e.g., “30 min estimated wait”), tourists can only make vague judgments based on their own psychological expectations or past experiences (Masiero & Qiu, 2018). In contrast, comparative framing provides a typical reference point. By introducing reference information, it changes the individual’s subjective evaluation of the current situation, so that tourists can compare the current waiting time with the others’ waiting times or historical waiting times. On the one hand, this framing can alleviate the anxiety and dissatisfaction generated during the waiting process, enabling tourists to view their current experience as a “reasonable wait” rather than a “passive loss”. On the other hand, comparative framing can activate the social comparative tendency of tourists (Ye et al., 2021). It not only provides an intuitive sense of comparative advantage, but also may reshape tourists’ perception of the value of the current wait (Dagogo-Jack, 2024). In other words, when tourists realize that their waiting time is better than that of others, they may obtain a sense of benefit and fairness, increasing the rationality of their decision-making process and thus improving their waiting intention. Therefore, this paper puts forward the following assumptions:

**H1:**  *Compared to non-comparative framing, the comparative framing of queuing time increases tourists’ waiting intention.*

### 3.2. The Mediating Role of Perceived Waiting Costs

When tourists evaluate whether to wait, they usually do not make rational calculations based solely on the objective length of time, but are significantly influenced by their subjective psychological perception (Huang & Chang, 2019). Perceived waiting cost is a key psychological variable in tourists’ waiting decisions. It not only includes a subjective assessment of the length of waiting time, but also reflects tourists’ comprehensive perception of opportunity loss, resource occupation, and psychological burden while waiting (Jo et al., 2025). Studies have shown that the perceived waiting cost significantly affects the service evaluation and behavioral willingness. The higher the cost, the more individuals tend to avoid waiting situations (Jo et al., 2025).

Comparative framing, as a vital external clue, can have an indirect impact on the waiting intention of tourists through affecting the perceived cost of waiting. Prospect theory notes that, when deciding, people avoid losses and seek significant similarities in their rewards (H. Zhang et al., 2023). By introducing reference points, comparative framing changes the cognitive baseline of tourists regarding waiting time, enabling tourists to view the current waiting time as a relative advantage or reasonable cost, and thereby reducing the subjective perception of waiting cost (Guo et al., 2025). This process can be put in line with the loss-aversion mechanism of prospect theory, where people tend to select more options that appear to minimize losses (Dogru et al., 2021). Furthermore, according to the mental accounting theory, individuals categorize experiences into separate psychological accounts with varying subjective values (Qiu et al., 2022). Tourists may classify their current waits under comparative framing as “low-cost” or a “fair exchange”, thereby reducing their sensitivity to time taken, opportunity losses, and emotional burden. This psychological reconstruction process helps tourists view waiting as an acceptable cost and enhances their waiting intention. Based on this, this paper proposes the following hypotheses:

**H2a:**  *Compared to non-comparative framing, the comparative framing of queuing time decreases tourists’ perceived waiting costs.*

**H2b:**  *Perceived waiting costs play a mediating role between comparative framing and tourists’ waiting intention.*

### 3.3. The Moderating Role of Queuing Settings

The type of queuing that tourists are considering itself constitutes a key situational characteristic, and the waiting environment affects an individual’s waiting perception (Ayodeji et al., 2023). Under the two modes of virtual queuing and physical queuing, there are significant differences in tourists’ attention distribution, sensory immersion degree, and dependence on temporal information, which affect their processing and response to information cues (Jo et al., 2025).

Cognitive load theory suggests that an individual’s cognitive resources are limited. In a high-load environment, individuals tend to prioritize information cues that are highly relevant to the current goal to regulate their cognitive burden (White & Willness, 2009). Physical queuing constitutes a high-load environment, in which tourists must remain in a confined space with limited freedom of movement, and their physical and mental attention is highly focused, resulting in strained cognitive resources (Zehrer & Raich, 2016; Yin et al., 2024b). Discomfort experienced during the waiting process is more pronounced, often inducing negative emotions such as anxiety and anger (S. Kim et al., 2016). In this context, tourists are more likely to attend to external information that can alleviate their discomfort, particularly cues about queue duration, to help assess the reasonableness of waiting. Therefore, when the queuing time is presented through a comparative framing, tourists are more likely to use this information to reevaluate the waiting cost, thereby enhancing their willingness to accept queuing. In contrast, virtual queuing grants tourists greater behavioral freedom. Individuals can move freely and distract themselves during the waiting period (Ryan & Valverde, 2006). Their attention to time information is significantly reduced, and their sensitivity to the contrast frame also weakens accordingly.

From a cognitive-processing perspective, in physical queuing, due to the more coercive waiting state, tourists need psychological compensation mechanisms more to alleviate traffic congestion, and their interpretive processing of time information is more active. Therefore, they are more susceptible to the influence of comparative framing (Grogans et al., 2023; Moore et al., 2025). In virtual queuing, tourists have a relative psychological distance towards time cues, and their processing may be more passive, or they may even ignore the content of the prompt (D.-H. Park, 2019). The psychological effects of “I’m waiting less than others”, triggered by comparative framing , will be more easily activated and transformed into behavioral motivation during physical queuing than in virtual queuing. The queuing settings in this path are not only a background variable but also serve as a moderating factor at the psychological processing level, influencing the intensity of the effect of the information-presentation method on the behavioral outcome. Based on this, the following hypotheses are proposed:

**H3:**  *The queuing settings moderate the effect of comparative framing on tourists’ waiting intention, and this impact is stronger in a physical queuing situation than in a virtual queuing situation.*

## 4. Overview of Studies

**Figure 1:**
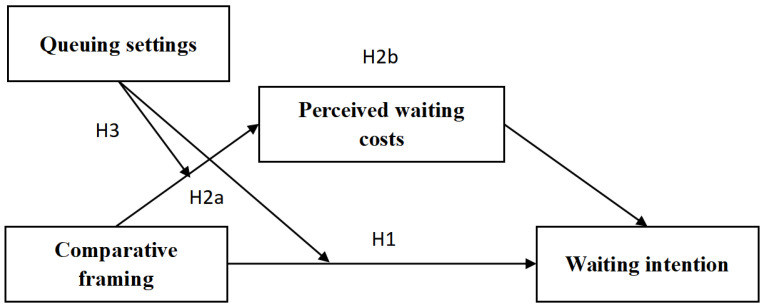
The conceptual model.

Three scenario-based experiments were conducted to test the proposed hypotheses (see Figure 1). Participants were invited online using the research platform Credamo, and were randomly distributed to the cases in the experiment. Study 1 aimed to verify the impact of information framing (comparative vs. non-comparative) on tourists’ waiting intention; 120 participants (50.0% female) were involved in this study. The purpose of Study 2 was to examine the mediating effect of perceived waiting costs, and another 120 subjects were used (52.5% male). On the basis of these results, Study 3 added the factor of queuing type (physical vs. virtual) as a moderator, with the aim of testing the relationship between queuing settings and the use of comparative framing to determine perceived waiting costs and waiting intention. Those who could not pass attention checks were excluded so as to obtain quality data. All three studies were collected in the period of August–October 2025. Power analysis and the power software G*Power (version 3.1.9.7) were used to compute suitable target samples in all studies to ensure sufficient statistical power (Faul et al., 2009). Detailed information about the experimental materials, measurement scales, and demographic information is presented in the Supplementary Materials (see Sections S1–S3).

## 5. Study 1

### 5.1. Sample and Procedures

This study employed a scenario-based experiment to examine the effect of framing (comparative vs. non-comparative) on tourists’ waiting intention. A total of 120 participants (50.0% female) were mainly recruited through an online experimental platform and randomly assigned to one of two conditions (comparative framing vs. non-comparative framing) using a between-subjects design. In the experimental task, the subjects read the description regarding queuing. To prevent the queuing time length influencing the results, three groups with queuing times within the normal range were randomly presented to the subjects, which were 20 min, 30 min, and 40 min (for specific case materials, see Section S3). Subsequently, the subjects completed questionnaires to measure waiting intention and the impact of the comparative framing, as well as collecting demographic information.

All measurements were conducted using a 5-point Likert scale (1 = strongly disagree; 5 = strongly agree), and existing mature scales were referenced to ensure the reliability and validity of the measurements. Tourists’ waiting intention was measured using three measures (Cronbach’s α = 0.813) (Jo et al., 2025).

### 5.2. Analysis and Results

An independent-samples t-test confirmed that the framing manipulation was successful: participants who received comparative framing scored significantly higher than those in the non-comparative framing group (M comparative framing = 4.85; M non-comparative framing = 1.22, t = 46.264, *p* < 0.01).

**Figure 2:**
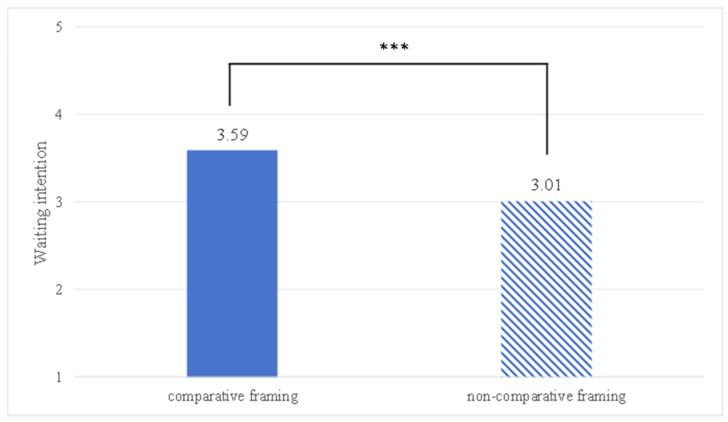
The influence of comparative framing on waiting intention; *** denotes *p* < 0.001.

The ANOVA results showed a significant main effect of framing on waiting intention. Tourists in the comparative framing group reported significantly higher waiting intention (M comparative framing = 3.59, SD = 0.97) than those in the non-comparative group (M non-comparative framing = 3.01, SD = 0.96), F (1, 118) = 10.758, *p* < 0.01; see Figure 2). Therefore, H1 was supported.

### 5.3. Discussion

Study 1 confirms the main effect of comparative framing regarding time on tourists’ waiting intention. However, this study only tested whether such framing affects waiting intention, without examining the underlying psychological processes. To address this limitation, Study 2 introduced perceived waiting costs as potential mediators, aiming to explore the psychological mechanisms underlying the effect of comparative framing regarding waiting time on tourists’ waiting intention.

## 6. Study 2

### 6.1. Sample and Procedure

Study 2 tested the mediating role of perceived waiting costs in the relationship between framing and waiting intention. A total of 120 participants (52.5% male) were recruited online and randomly assigned to either a comparative or non-comparative framing condition. After reading the scenario, participants completed measures focused on waiting intention, perceived waiting costs, comparative framing, and demographics.

Consistent with the economic theory of time, customers primarily evaluated waiting in terms of how much time is lost and what alternative activities are forgone (Leclerc & Schmitt, 1999). Perceived waiting costs were assessed using two dimensions. Perceived wait time was measured with three items developed by S. Li et al. (2021), and perceived opportunity cost was measured with three items developed by Jo et al. (2025). All measures demonstrated strong reliability: waiting intention (Cronbach’s α = 0.876), perceived wait time (Cronbach’s α = 0.822), and perceived opportunity cost (Cronbach’s α = 0.854).

### 6.2. Analysis and Results

Participants in the comparative framing group reported significantly higher scores than those in the non-comparative group (M comparative framing = 4.82 vs. M non-comparative framing = 1.25, t = 39.938, *p* < 0.01). Thus, the manipulation check was successful.

The ANOVA results revealed a significant main effect of comparative framing. Participants exposed to comparative framing reported higher waiting intention (M comparative framing = 3.58, SD = 0.68) than those who experienced the non-comparative condition (M non-comparative framing = 2.83, SD = 0.98), F (1, 118) = 23.887, *p* < 0.01). Significant group differences were also found in perceived wait time (M comparative framing = 2.79; M non-comparative framing = 3.57, F (1, 118) = 27.583, *p* < 0.01) and perceived opportunity cost (M comparative framing = 2.90; M non-comparative framing = 3.66, F (1, 118) = 16.224, *p* < 0.01). Thus, H1 and H2a were supported.

**Figure 3:**
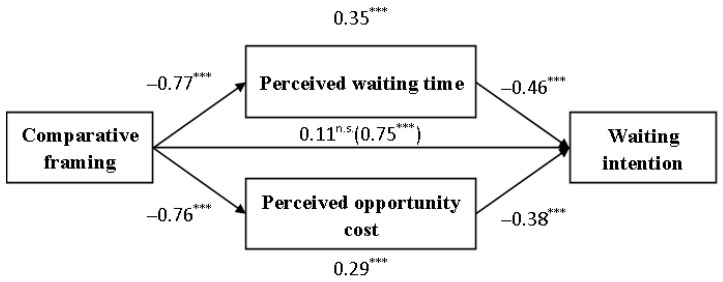
Mediating effect in Study 2. Note: *** *p* < 0.001; ^n.s.^ *p* > 0.05.

A mediation analysis using a PROCESS Model 4 with 5000 bootstrap samples was conducted, with framing condition coded as 0 = non-comparative framing and 1 = comparative framing. The results indicated that both perceived wait time (β = 0.35, SE = 0.10, 95% CI [0.1888, 0.5545]) and perceived opportunity cost (β = 0.29, SE = 0.10, 95% CI [0.1188, 0.5059]) significantly mediated the relationship between comparative framing and waiting intention. The total indirect effect was significant (β = 0.64, SE = 0.13, 95% CI [0.3854, 0.9054]). The direct effect was non-significant (β = 0.11, SE = 0.09, 95% CI [−0.0729, 0.2874], see Figure 3). Therefore, H2b was supported.

### 6.3. Discussion

Study 2 provides evidence that the effect of comparative framing on tourists’ waiting intention is mediated by perceived waiting costs, including both perceived wait time and perceived opportunity costs. However, Study 2 did not account for variations in queuing settings. Since tourists’ attention and emotional responses can differ depending on the type of queue, it remains unclear whether the observed effects are consistent across the different waiting contexts. Therefore, Study 3 introduced queuing setting as a moderator to explore whether the effects of comparative framing vary between physical and virtual queues.

## 7. Study 3

### 7.1. Sample and Procedure

Study 3 tested whether queuing settings moderated the effect of comparative framing on waiting intention. A total of 240 participants (52.5% male) were recruited and randomly assigned to one of four conditions. After completing the scenario-based task, participants answered questions regarding their waiting intention, perceived waiting costs, comparative framing, queuing settings, and demographics.

All scales demonstrated high reliability: waiting intention (Cronbach’s α = 0.849), perceived wait time (Cronbach’s α = 0.897), and perceived opportunity cost (Cronbach’s α = 0.903).

### 7.2. Analysis and Results

Manipulation checks confirmed that both comparative framing and queuing setting manipulations were successful. Participants in the comparative framing group scored significantly higher than those in the non-comparative group (M comparative framing = 4.82 vs. M non-comparative framing = 1.24, t = 53.418, *p* < 0.01). Those in the virtual queue condition also scored significantly higher than those in the physical queue condition (M virtual queue = 4.88 vs. M physical queue = 1.15, t = 78.172, *p* < 0.01).

The ANOVA results showed a significant main effect of comparative framing on waiting intention: comparative framing (M comparative framing = 3.83, SD = 0.97) led to higher willingness than non-comparative framing (M non-comparative framing = 3.45, SD = 1.10, F (1, 238) = 7.697, *p* < 0.01). Comparative framing also had significant effects on perceived wait time (M comparative framing = 2.16; M non-comparative framing = 2.59, F (1, 238) = 10.113, *p* < 0.01) and perceived opportunity cost (M comparative framing = 2.24; M non-comparative framing = 2.61, F (1, 238) = 6.193, *p* = 0.01). Thus, H1 and H2a were supported.

Mediation analysis (PROCESS Model 4) showed that both perceived wait time (β = 0.20, SE = 0.07, 95% CI [0.0742, 0.3384]) and perceived opportunity cost (β = 0.17, SE = 0.07, 95% CI [0.0351, 0.3061]) significantly mediated the effect of comparative framing on waiting intentions. The total indirect effect was significant (β = 0.37, SE = 0.13, 95% CI [0.1205, 0.6222]). The direct effect was not significant (β = 0.01, SE = 0.05, 95% CI [−0.0869, 0.0944]), supporting H2b.

**Figure 4:**
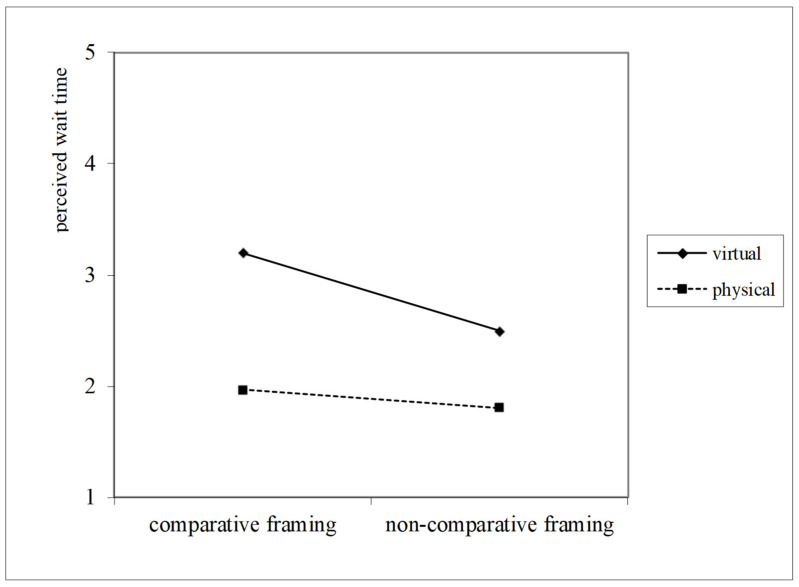
Queuing settings moderate the effect of comparative framing on perceived wait time, showing a stronger reduction in physical queues.

**Figure 5:**
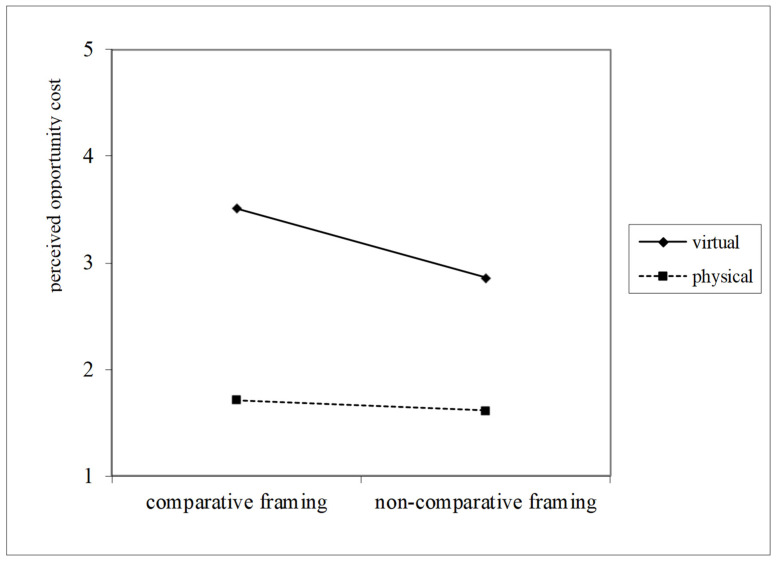
Queuing settings moderate the effect of comparative framing on perceived opportunity cost, showing a stronger reduction in physical queues.

A moderated mediation analysis was conducted using PROCESS Model 8 with 5000 bootstrap samples and 95% confidence intervals, with queuing setting coded as 0 = physical queue and 1 = virtual queue. The index of moderated mediation was significant for the path via perceived opportunity cost (β = −0.16, SE = 0.07, 95% CI [−0.3076, −0.0296]) and for the path via perceived wait time (β = −0.30, SE = 0.13, 95% CI [−0.5560, −0.0368]). In physical queues, comparative framing had significant indirect effects on waiting intention via perceived opportunity cost (β = 0.18, Boot SE = 0.06, 95% CI [0.0802, 0.3204]) and perceived wait time (β = 0.39, SE = 0.08, 95% CI [0.2193, 0.5465]). In contrast, in virtual queues, the indirect effects via perceived opportunity cost (β = 0.03, SE = 0.05, 95% CI [−0.0554, 0.1229]) and perceived wait time (β = 0.09, SE = 0.10, 95% CI [−0.1134, 0.2908]) were not significant. Therefore, H3 was supported (See Figure 4 and Figure 5).

### 7.3. Discussion

Study 3 extended the findings by examining the moderating role of queuing settings. The results show that the effect of comparative framing on perceived waiting costs and waiting intention is significantly stronger in physical queues than in virtual queues. These findings suggest that the impact of the framing depends not only on how information is presented but also on the specific characteristics of the waiting context. Together, the three studies offer a more comprehensive understanding of the psychological mechanisms and boundary conditions of comparative framing in shaping tourists’ waiting decisions.

## 8. Conclusions

This study systematically examines the influence mechanism of comparative framing on tourists’ waiting intention in queuing situations. Study 1 first verified the basic effect of comparative framing. The results show that, compared with non-comparative framing, comparative framing can effectively improve tourists’ waiting intention. On this basis, Study 2 further introduced perceived waiting cost as an intermediary variable. The results found that comparative framing can significantly reduce tourists’ perceived waiting costs, thus indirectly improving their waiting intention. Study 3 comprehensively examined the moderating effect of queuing setting, and revealed the boundary conditions of the contrast framing effect by manipulating physical queuing and virtual queuing situations. The results show that the influence of comparative framing in the physical queuing situation is significantly stronger than in virtual queuing, indicating that there are differences in the psychological reference point sensitivity of tourists in different queuing settings. In summary, through these three experiments, this article reveals the psychological mechanism and situational boundaries of using comparative framing to improve tourists’ waiting intention, expands the application of prospect theory in tourism service scenarios, and provides guidance for peak queuing management and service information optimization.

## 9. Discussion

### 9.1. Theoretical Contributions

Firstly, this study has broadened the theoretical horizon of the research focus on the queuing behavior of tourists as the focus of analysis was shifted away from waiting costs and placed on the framing and presentation of information about the waiting costs. Earlier research mainly explored how perceived waiting costs and predictability impact tourist satisfaction and their intentions to behave, how tourists apply service evaluations, and how social comparison or normative reference cues can influence service evaluation (Ayodeji & Rjoub, 2021; Qin et al., 2023; Böddeker et al., 2025). Conversely, the comparative framing used in this study is conceptualized as an information-level cognitive framing process, as opposed to an interpersonal comparison or norm-based benchmarking. The comparative references in our design are inherent in the presentation of waiting cost information and are not dependent on the existence of other consumers, social norms, or performance standards.

Secondly, this study identified the key mediating role of perceived waiting costs in the influence of comparative framing on tourists’ behavioral intentions, deepening the understanding of the psychological mechanism of queuing behavior. Under general consumption conditions, existing research presents comparisons as a means of affecting user assessment and behavioral choices (Dahl et al., 2012), frequently focusing on social comparisons involving individuals or the perceived distinctiveness that comes as a result of product comparisons. In contrast, this paper highlights the role of comparative framing as one of the cognitive processes that can reshape tourists’ subjective perception of the costs of waiting. The reactions of the tourists towards waiting are thus not objective responses to the time and amount of effort being spent but subjective reactions responding to cognitive processes and the subjective importance of the perceived costs.

Thirdly, the research reveals the moderating role of queuing settings in the influence path of comparative framing, enriching the exploration of boundary conditions in information-presentation effects. Specifically, the research found that the effect of comparative framing in the physical queuing context is significantly stronger than that in the virtual queuing context. This result indicates that tourists are more dependent on positive information cues to regulate their emotions and make decisions in physical queuing, where their bodies are occupied and their attention is limited. This finding not only supplements the external validity of the framing effect in tourism scenarios, but also echoes the classic discussion of cognitive load theory regarding the depth of information-processing.

### 9.2. Practical Implications

Firstly, the research shows that, compared with presenting only the absolute waiting time, presenting information through comparative framing can significantly enhance tourists’ waiting intention. This implies that tourist attractions, theme parks, and other peak congestion sites do not necessarily need to increase manpower or substantially reduce the actual waiting cost. Instead, they can effectively alleviate tourists’ dissatisfaction and potential attrition risks by optimizing how waiting cost information is communicated. For tourism operators with limited resources but strong service pressure, this information design strategy has a strong cost–effectiveness ratio.

Secondly, this research indicates that perceived waiting costs are a core psychological variable shaping tourists’ behavioral reactions, implying that attempts to optimize the queuing experience should place greater emphasis on tourists’ subjective experience. Particular indicators of such measures include the proactive provision of waiting-time comparison information on large screens, on boards located in the form of posters, or through using mobile devices, as well as emotional comfort alerts to allow tourists to maintain a positive psychological state and become less sensitive to the waiting costs. This design, based on psychological expectation management, can increase the overall assessment of tourists’ experience in high-flow situations.

Thirdly, the study found that the effectiveness of the information presentation is significantly affected by the queuing setting, with tourists in physical queues being more sensitive to waiting-cost-related information. This result provides a more targeted management strategy for tourist destinations or service institutions. For example, in on-site physical queuing situations, managers can strengthen the visual design and increase the frequency of comparative waiting cost cues. In virtual queues or online booking systems, more flexible information designs that align with tourists’ freedom of movement may be more effective. By matching different queuing scenarios and information strategies, the coordinated optimization of situational awareness and psychological guidance can be realized in service management.

### 9.3. Limitations and Future Research

First, the current research mainly examines the mediating role of perceived waiting costs and the moderating role of queuing settings, and fails to include the personal characteristics of tourists (such as their time-sensitivity, information-processing style, or service expectations). Future research could combine individual differences and personality variables to further explore which tourists are more susceptible to the influence of comparative information to enrich the individual psychological portraits when examining queuing behavior.

Second, a scenario-based experiment was used in this study, which aids in explaining the causal processes behind the perception of waiting costs. The method has weaknesses, however, when it comes to external validity. Notably, the experimental conditions could not capture the intricate conditions which tourists encounter when queuing at peak times, i.e., social engagements, emotional swings, or other undesirable on-site influences. It is also important to point out that this study is based on the waiting intention of tourists and not their actual queuing behavior; hence, attempts to interpret their actual behavior should be made cautiously. In addition, the manipulations in the experiments were rather open, and participants could have perceived the purpose of the experiments. This creates a risk of demand characteristics that might have affected the behavior of the participants and increased the effect that was measured. Further studies could undertake field tests in the real tourist sites to examine the validity and strength of the model in the natural environments. For example, displaying various waiting cost details or comparative reference signs to visitors at peak times and documenting their real queuing behavior might be effective in diminishing the effect of demand nature.

Third, this study adopts a static perspective, which fails to reveal the dynamic psychological changes in tourists during the process of queuing. Future research may use multi-time-point tracking or in situ multi-experimental designs in order to gather information about the perceived waiting costs and the perceived behavioral intentions of tourists at various times during the queuing process. Such a methodology would increase the dynamic nature of the waiting experience and offer a richer source of empirical evidence regarding the dynamic nature of waiting intention over time with respect to various types of queuing or various information-presentation strategies.

## Data Availability

The original contributions presented in this study are included in the article. Further inquiries can be directed to the corresponding author.

## Supplementary Materials

The following supporting information can be downloaded at: https://www.mdpi.com/article/10.3390/bs16020167/s1, Figure S1: Experimental materials for the comparative framing group; Figure S2. Experimental materials for the non-comparative framing group; Table S1: Demographic characteristics of the participants; Table S2: Measurement scales.
