# Supplementary material for: Worth the Wait? The Effect of Comparative Framing on Tourists’ Waiting Intention
Source: Behav Sci (Basel). 2026 Jan 25;16(2):167. doi: 10.3390/bs16020167 (PMC12937780; doi:10.3390/bs16020167)
Supplement: Supplementary file 1 [file behavsci-16-00167-s001.zip › behavsci-4048532-supplementary.pdf]

Supplementary Section S1. A summary of participants' demographic information.

Table S1. Demographic characteristics of the participants.

	Study1		Study2		Study3	
	n	%	n	%	n	%
Age						
18 to 25	49	40.8%	48	40.0%	84	35.0%
26 to 35	35	29.2%	48	40.0%	92	38.3%
36 to 45	27	22.5%	17	14.2%	45	18.8%
46 to 60	8	6.7%	4	3.3%	18	7.5%
61 and older	1	0.8%	3	2.5%	1	0.4%
Gender						
Female	60	50.0%	57	47.5%	114	47.5%
Male	60	50.0%	63	52.5%	126	52.5%
Education						
High school or below	12	10.0%	13	10.8%	16	6.7%
3-year College	30	25.0%	17	14.2%	40	16.7%
4-year University	57	47.5%	66	55.0%	119	49.6%
Postgraduate	21	17.5%	24	20.0%	65	27.1%
Occupation						
Worker	12	10.0%	6	5.0%	19	7.9%
Public servant	10	8.3%	17	14.2%	44	18.3%
Enterprise staff	47	39.2%	51	42.5%	86	35.8%
Student	39	32.5%	36	30.0%	73	30.4%
Other	12	10.0%	10	8.3%	18	7.5%
Monthly Income (USD)						
≤421	19	15.8%	23	19.2%	27	11.3%
422-703	24	20.0%	30	25.0%	47	19.6%
704-984	32	26.7%	31	25.8%	82	34.2%
985-1406	41	34.2%	27	22.5%	65	27.1%
≥1407	4	3.3%	9	7.5%	19	7.9%

(Note: According to the exchange rate on that day, 1 US dollars equaled 7.11 Chinese yuan (RMB). The original scale items were ≤3000, 3001－4999, 5000－6999, 7000－9999, ≥10,000 RMB.)

Supplementary Section S2. Measurement scales.

Table S2. Measurement scales.

Variable	Item (Five-point Likert scale)	Reference
Waiting intention	I will stay in a queue for the theme park attraction.	(Jo et al., 2025)
	I am willing to wait to be seated in the theme park attractiont.	
	I am planning to wait for the theme park attraction.	

Perceived wait time	Overall, I would say my waiting time for this theme park attraction was long<br>Overall, I would say my waiting time for this theme park attraction was unacceptable<br>Overall, I would say my waiting time for this theme park attraction was unreasonable	(Li et al., 2021)
Perceived opportunity cost	The waiting for this theme park attraction will delay my plan.<br>The waiting for this theme park attraction will make me lose other options.<br>The waiting time for this theme park attraction will force me to postpone other plans.	(Jo et al., 2025)

Supplementary Section S3. Experimental materials

Experimental materials of Study

You are visiting a theme park, and Attraction A is one of the rides you are most interested in. When you check the park's map app, you find that the current waiting time for this attraction is 40 minutes.

Comparative framing

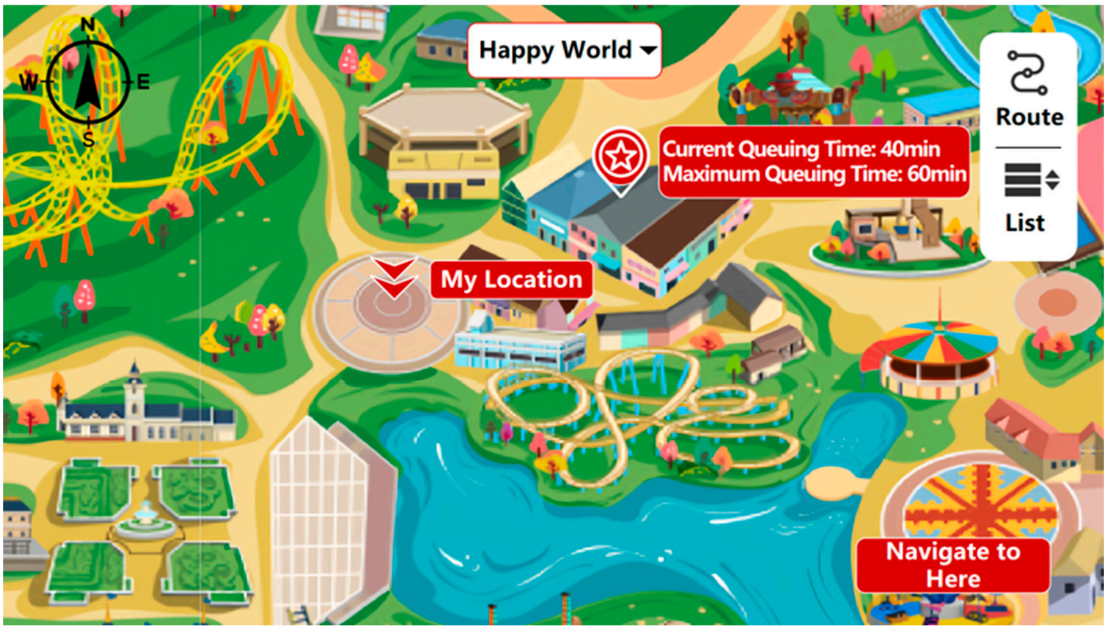

Figure S1. Experimental materials for the comparative framing group.

Non-comparative framing

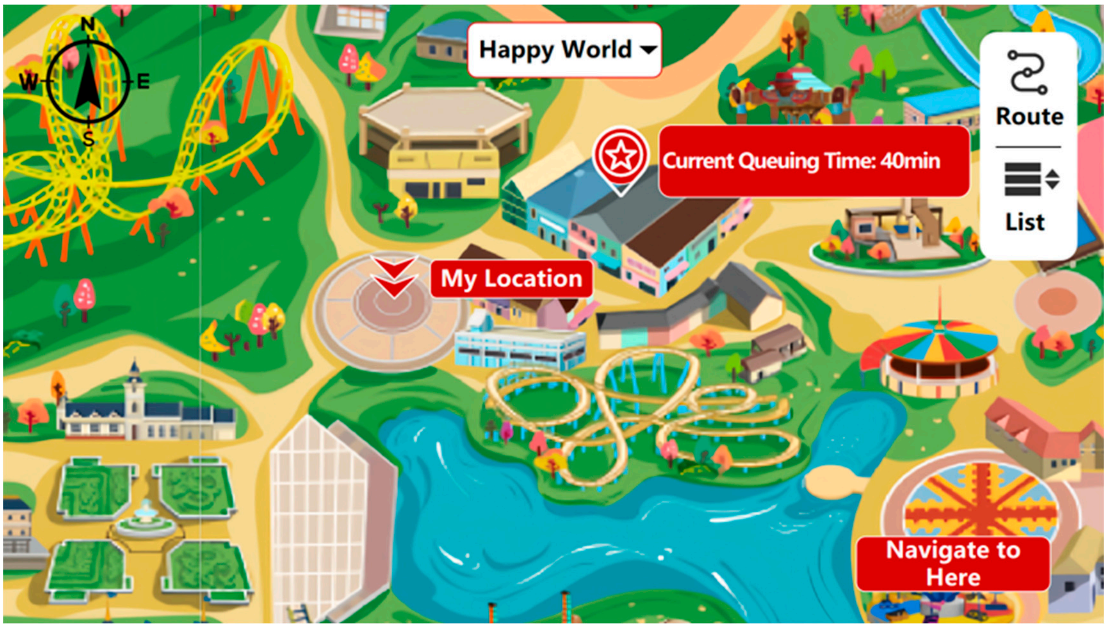

Figure S2. Experimental materials for the non-comparative framing group.
